# Design and Characterization of Curcumin-Modified Polyurethane Material with Good Mechanical, Shape-Memory, pH-Responsive, and Biocompatible Properties

**DOI:** 10.3390/biom15081070

**Published:** 2025-07-24

**Authors:** Man Wang, Hongying Liu, Wei Zhao, Huafen Wang, Yuwei Zhuang, Jie Yang, Zhaohui Liu, Jing Zhu, Sichong Chen, Jinghui Cheng

**Affiliations:** 1 High & New Technology Research Center of Henan Academy of Sciences, No. 56 Hongzhuan Road, Zhengzhou 450002, China; zhaowei@hnas.ac.cn (W.Z.); wanghuafen_2025@hnas.ac.cn (H.W.); yuweizhuang0218@163.com (Y.Z.); yangjie@hnas.ac.cn (J.Y.); liuzhaohui@hnas.ac.cn (Z.L.); zjlsl7443@hnas.ac.cn (J.Z.); 2 West China School of Nursing, Sichuan University, Chengdu 610041, China; liuhongying@scu.edu.cn; 3 The Collaborative Innovation Center for Eco-Friendly and Fire-Safety Polymeric Materials (MoE), National Engineering Laboratory of Eco-Friendly Polymeric Materials (Sichuan), College of Chemistry, State Key Laboratory of Polymer Materials Engineering, Sichuan University, Chengdu 610064, China; chensichong@scu.edu.cn; 4 Key Laboratory of Chemo/Biosensing and Detection of Xuchang, Key Laboratory of Micro-Nano Materials for Energy Storage and Conversion of Henan Province, Henan Joint International Research Laboratory of Nanomaterials for Energy and Catalysis, College of Chemical and Materials Engineering, Xuchang University, Xuchang 461000, China

**Keywords:** polyurethane, curcumin, shape memory, pH responsiveness, biocompatibility

## Abstract

In the context of critical challenges in curcumin-modified polyurethane synthesis—including limited curcumin bioavailability and suboptimal biodegradability/biocompatibility—a novel polyurethane material (Cur-PU) with good mechanical, shape memory, pH-responsive, and biocompatibility was synthesized via a one-pot, two-step synthetic protocol in which HO-PCL-OH served as the soft segment and curcumin was employed as the chain extender. The experimental results demonstrate that with the increase in Cur units, the crystallinity of the Cur-PU material decreases from 32.6% to 5.3% and that the intensities of the diffraction peaks at 2θ = 21.36°, 21.97°, and 23.72° in the XRD pattern gradually diminish. Concomitantly, tensile strength decreased from 35.5 MPa to 19.3 MPa, and Shore A hardness declined from 88 HA to 65 HA. These observations indicate that the sterically hindered benzene ring structure of Cur imposes restrictions on HO-PCL-OH crystallization, leading to lower crystallinity and retarded crystallization kinetics in Cur-PU. As a consequence, the material’s tensile strength and hardness are diminished. Except for the Cur-PU-3 sample, all other variants exhibited exceptional shape-memory functionality, with R_f_ and R_r_ exceeding 95%, as determined by three-point bending method. Analogous to pure curcumin solutions, Cur-PU solutions demonstrated pH-responsive chromatic transitions: upon addition of hydroxide ion (OH^−^) solutions at increasing concentrations, the solutions shifted from yellow-green to dark green and finally to orange-yellow, enabling sensitive pH detection across alkaline gradients. Hydrolytic degradation studies conducted over 15 weeks in air, UPW, and pH 6.0/8.0 phosphate buffer solutions revealed mass loss <2% for Cur-PU films. Surface morphological analysis showed progressive etching with the formation of micro-to-nano-scale pores, indicative of a surface-erosion degradation mechanism consistent with pure PCL. Biocompatibility assessments via L929 mouse fibroblast co-culture experiments demonstrated ≥90% cell viability after 72 h, while relative red blood cell hemolysis rates remained below 5%. Collectively, these findings establish Cur-PU as a biocompatible material with tunable mechanical properties, and pH responsiveness, underscoring its translational potential for biomedical applications such as drug delivery systems and tissue engineering scaffolds.

## 1. Introduction

Curcumin (Cur) is a natural compound with effects including lipid-lowering, anti-tumor, anti-inflammatory, choleretic, and antioxidant activities [1,2,3,4,5,6]. Clinical studies have shown that Cur can inhibit the carcinogenesis of a variety of cells, including breast cancer, gastric cancer, liver cancer, leukemia, oral epithelial cancer, ovarian cancer, pancreatic cancer, prostate cancer, etc. It can also induce apoptosis in cancer cells while exerting no toxic effects on healthy cells. Therefore, Cur has emerged as a potential compound for the development of anticancer drugs [7]. Curcumin is a relatively rare pigment with a diketone structure in the plant kingdom. The diketone in its structure can form metal chelates with copper and iron. This not only enhances the pharmacological activity of Cur but also reduces the toxicity of metal ions. In addition, Cur, as a commonly used natural pigment, is widely applied in the food industry, mainly for the coloring of sausage, braised, and marinated products, thanks to its outstanding properties [8,9,10].

Polyurethane is a novel polymeric material synthesized through the reaction between polyisocyanates and hydroxyl-terminated compounds. By virtue of its outstanding mechanical properties, remarkable wear resistance, and high resilience, polyurethane finds extensive applications in multiple fields, including coatings, adhesives, elastomers, and foam materials [11,12,13]. Among various types of polyurethane, the type prepared from biodegradable polymer precursors not only demonstrates excellent mechanical properties and favorable processability but also exhibits certain biodegradability and good biocompatibility. As a result, it holds significant application prospects in fields like biomedical materials [14,15]. However, polyurethane materials also have some drawbacks. For instance, their biodegradability is limited, and their additional biological functions are rather singular, which restricts their applications in the biomedical field. If Cur is introduced into the polyurethane structure, either through intrinsic introduction or surface modification, the complementary advantages of curcumin and polyurethane materials can be realized. This enables the development and synthesis of a new-type biomedical polyurethane material, endowing polyurethane materials with multifunctionality. Notably, Cur shows antioxidant, anticancer, and antibacterial properties due to the presence of terminal hydroxyl groups and enol isomers within the molecule. The hydrogen atoms can be transferred to the diketone chain of Cur, allowing the functional groups (hydroxyl groups, double bonds, benzene rings, and diketone structures) connected to Cur to play a crucial role in its activity. Cur can participate in major chemical reactions related to its biological activity, including hydrogen oxidation, Michael reaction, hydrolysis, degradation, and enzymatic reaction. By modifying polyurethane materials with curcumin, structurally stable biomedical polyurethane materials can be obtained, while possessing the excellent properties of both, achieving complementary advantages and holding great promise for preparation of high-performance and multifunctional biomedical polyurethane materials.

Cur confronts several key challenges. Its poor water solubility; proneness to decomposition; and high sensitivity to light, temperature, and ultraviolet light pose serious threats to its biological activity. These issues not only attenuate its antibacterial and antioxidant capabilities but also restrict its medical efficacy in clinical applications, as documented in References [16,17]. Currently, the literature has reported multiple strategies to enhance its water solubility and bioavailability. These methods involve encapsulation within polymer micelles, liposomes, polymer nanoparticles, lipid-based nanoparticles, and hydrogels, as detailed in References [18,19,20,21,22,23]. For instance, Song [24] synthesized curcumin-loaded PLGA-PEG-PLGA triblock copolymeric micelles, which effectively enhanced the bioavailability of curcumin. Lin [25] prepared Curcumin β-Cyclodextrin Inclusion Complex, Curcumin Solid Dispersion, and Curcumin Phospholipid Complex to boost the bioavailability of curcumin. Satoshi [26] noted that polyvinylpyrrolidone (PVP) and vinylpyrrolidone-vinyl acetate copolymers with a pyrrolidone skeleton held promise for increasing the solubility of curcumin in water. Miao [27] fabricated a multifunctional bacterial cellulose (BC)-based film (termed BC-PE-Cur) for intelligent food packaging. Yang [28] developed curcumin gelatin nanoparticles with brain tissue-targeting effects (Cur@GAR NPS), improving the solubility of curcumin. Huang [29] prepared a nanohybrid hydrogel patch composed with natural-derived substances (curcumin@tannic acid (Cur@TA) nanoparticles). This patch exhibits dual functionalities of chemically scavenging ROS and physically absorbing radiation, thereby alleviating radiation damage.

Both surface ion treatment and solution immersion grafting of Cur represent methods for surface modification of polyurethane materials with Cur. Despite the successful introduction of Cur onto the surface of polyurethane materials, the interaction between Cur and polyurethane is feeble, and the bioavailability of Cur is inadequate. This situation renders it arduous to exert the ideal biological efficacy. To further enhance the bioavailability of Cur within polyurethane materials, Kashif Mahmood et al. [30] synthesized polyurethanes with excellent antibacterial properties using Cur and BDO as dual chain extenders. The authors investigated the effects of varying the diisocyanate structure on the surface morphology, degree of phase separation, and antibacterial properties of curcumin-based polyurethanes. However, since the authors employed BDO and Cur as dual chain extenders, the incorporation amount of curcumin was limited, and thus, the influence of curcumin content on the properties of polyurethanes has not been studied in detail.

**Scheme 1:**
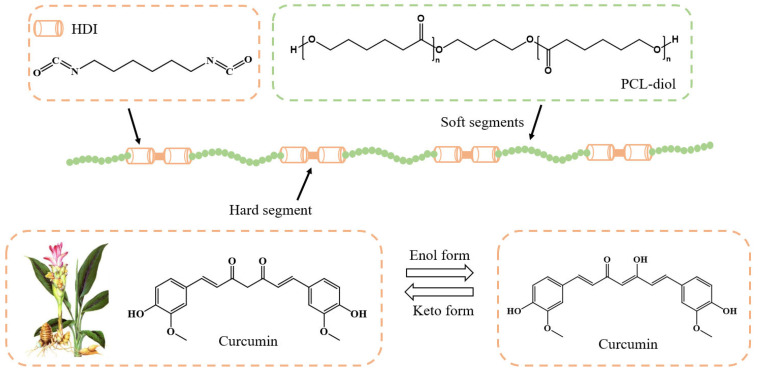
The chemical structures of curcumin-modified polyurethane in this work.

In this paper, we intend to synthesize a polyurethane material (Cur-PU) with excellent mechanical properties and good biocompatibility using HO-PCL-OH as the soft segment and curcumin as the sole chain extender. This approach can not only improve the bioavailability of curcumin but also enable precise regulation of the thermal properties, crystallization properties, mechanical properties, hydrolytic degradability, and biocompatibility of the polyurethane material by adjusting the curcumin content, thereby achieving the customized synthesis of biomedical polyurethane materials (as shown in Scheme 1).

## 2. Materials and Methods

### 2.1. Materials

The ε-caprolactone (ε-CL) monomer, purity ≥ 99%) was supplied by J&K Chemical (Beijing, China). Curcumin (analytical reagent, AR, ≥ 98%), hexamethylene diisocyanate (HDI ≥ 99%), N,N-Dimethylformamide (DMF ≥ 99.8%, Water ≤ 50 ppm (by Karl Fischer titration, MkSeal 500 mL), sodium hydroxide (NaOH, AR), phosphoric anhydride (P_2_O_5_, >98.5%), 1,4-butanediol (BDO ≥ 99%), anhydrous sodium phosphate dibasic (Na_2_HPO_4_ ≥ 99%), and Sodium Phosphate Monobasic (NaH_2_PO_4_ ≥ 99.9%) were purchased from Macklin (Shanghai, China). Bisdemethoxycurcumin (≥98%, RG), stannous octoate (Sn(Oct)_2_, purity 92.5–100%), and hexafluoroisopropanol (HFIP, AR) were obtained from Adamas (Shanghai, China), Aladdin Chemical (Shanghai, China), and Tansoole (Shanghai, China), respectively. Methanol (MeOH, AR) and chloroform (CHCl_3_, AR) were procured from Kelong Reagent Corp. (Chengdu, China) and used without further purification. Prior to use, ε-CL monomer and BDO were purified via vacuum distillation over calcium hydride (CaH_2_). Curcumin and bisdemethoxycurcumin were dried in an oven at 40 °C to constant weight before use.

### 2.2. Methods

Nuclear Magnetic Resonance (NMR) spectra were recorded in *d*_6_-DMSO using a 400 MHz Agilent Technologies spectrometer operated at room temperature (Santa Clara, CA, USA). Chemical shifts (δ) for ^1^H were referenced to the tetramethylsilane (TMS) internal standard (0.00 ppm) and the *d*_6_-DMSO solvent peak (2.50 ppm), respectively. Fourier-transform infrared (FTIR) spectra were collected on Nicolet 6700 spectrometer (Thermo Fisher Scientific, Waltham, MA, USA) in the wavenumber range of 600~4000 cm^−1^. Gel permeation chromatography (GPC) was performed on an HLC-8320 system (Tosoh Corporation, Tokyo, Japan) equipped with a refractive index detector. DMF was used as the eluent at a flow rate of 1 mL min^−1^ and a column temperature of 30 °C. Thermogravimetric analysis was conducted on a TG STA 2500 Regulus thermogravimetric analyzer (NETZSCH, Selb, Germany) under a nitrogen flow of 50 mL min^−1^. Samples were heated from 40 to 700 °C at a heating rate of 10 °C min^−1^. Differential scanning calorimetry (DSC) measurements were carried out using a TA DSC25 instrument (TA Instruments, USA) in an aluminum pan under N_2_ flow of 50 mL min^−1^. The thermal protocol was as follows: the samples were first heated to 80 °C at 5 °C min^−1^ and held for 2 min to eliminate thermal history, then cooled to −80 °C at a 5 °C min^−1^, and finally reheated to 80 °C at the same rate. The crystallinity index (χ) of PCL was calculated using the following equation:

$$\chi \left(c,PCL\right)\left(\%\right)=\frac{{∆H}_{m,pcl}}{{∆H}_{0,pcl}\;\times \varphi }\times 100\%$$

where ∆H_m,pcl_ is the experimental melting enthalpy, and φ is the weight fraction of the corresponding component in the blend. ∆H_0,PCL_ = 139 J/g for PCL was used according to the reported enthalpy of the melting of 100% crystalline PCL [31].

Mechanical properties of samples were evaluated using a Gotech Testing Machines (Gotech AI-7000-M, Guangzhou, China) at a crosshead speed of 10 mm min^−1^, at room temperature. Each sample was measured three times, and the average value was taken. X-ray diffraction (XRD) patterns were acquired on a Bruker AXS D8 Advance diffractometer (Bruker, Karlsruhe, Germany) using Cu Kα radiation (λ = 0.15418 nm) at 40 kV and 5 mA. Scans were performed in the 2θ range of 5–50°, with a step size of 0.02° and a scanning rate of 5° min^−1^. Scanning electron microscopy (SEGMA, Baden-Württemberg, Germany) was used to observe the surfaces morphology of specimens. UV/Vis absorption spectra were recorded on a Shimadzu UV-3600 Plus spectrophotometer (Shimadzu, Kyoto, Japan) using 1 cm pathlength quartz cuvettes.

### 2.3. The Shape-Memory Experiment

The shape-memory experiment of Cur-PU films was conducted using the three-point bending method. Firstly, rectangular Cur-PU splines were placed in an oven at 60 °C for 5 min to maintain a constant temperature. The splines were then folded, and the temperature was rapidly reduced to 25 °C under a certain pressure to fix the temporary shape (θ_0_). Finally, the splines with the temporary shape were replaced in a constant-temperature water bath at 60 °C for recovery, and the recovered shape (θ) was obtained. The shape-fixity ratio (R_f_) and shape-recovery ratio (R_r_) in the shape-memory test are calculated using Formula (1), where θ and θ_0_ represent the angle size of the spline after fixation and recovery, respectively. Each sample was measured three times, and the average value was taken.

(1) $$R_{f}=\frac{180-\theta_{0}}{180}\times 100\%\;\;R_{r}=\frac{\theta -\theta_{0}}{180}\times 100\%$$

### 2.4. Degradation Experiment

Polymer films (100 mm×100 mm×0.5 mm) for degradation testing were prepared by compression molding at 70 °C for 5 min, under a pressure of 10 MPa. The films were subsequently cut into square specimens (10 mm × 10 mm × 0.5 mm) to measure mass loss during degradation.

Degradation tests were conducted using a 100 mL silk reagent bottle in a controlled environment chamber set at 25 °C and 60% relative humidity. For the air, UPW, and pH 6.0/8.0 phosphate buffer-solutions medium, the solution was replaced every week. At the end of each predetermined incubation period, the supernatant was decanted, and the remaining specimen was thoroughly washed thoroughly with deionized water, dried under vacuum at room temperature for three days to a constant weight, and then weighed to record the mass loss before and after degradation, calculated using Formula (2) [32]. (W_0_: the mass of the sample before degradation; W_i_: the mass of the sample after degradation.)

(2) $$Mass\;loss\left(\%\right)=\frac{W_{i}-W_{0}}{W_{0}}\times 100\%$$

### 2.5. Cytotoxicity Test

Specimens (Cur-PU-1, Cur-PU-2, Cur-PU-3, Cur-PU-4, and BDO-PU) were immersed in DMEM supplemented with 10% PBS and incubated for 24 h under 5% CO_2_ at 37 °C. L929 mouse fibroblasts were seeded into 96-multiwell plates at a density of 5 × 10^4^ cells per well in 500 μL of 10% PBS DMEM and cultured for 24 h at 37 °C. The old culture medium was then aspirated, and 200 µL of extract from each specimen group was added. The plates were further incubated for 24 h, 48 h, and 72 h under the same conditions, after which cell viability was evaluated using a CCK-8 assay [33]. Each sample was measured in triplicate, and the average value was finally obtained, with error bars recorded.

### 2.6. Hemolysis Assay

Rat blood (1 mL) was centrifuged at 3000 rpm for 10 min, and the upper serum was discarded. The red blood cell pellet was washed three times with PBS. Subsequently, 20 µL of RBCs was mixed with 500 µL of PBS extract containing different Cur-PU samples. Ionized water and PBS were used as positive and negative controls, respectively. The mixtures were incubated at 37 °C for 4 h, and then centrifuged at 3000 rpm for 10 min. The absorbance of hemoglobin in the supernatant was measured at 540 nm, using a UV-Vis spectrophotometer, and the hemolysis rate (HR) was calculated using Formula (3) [34].

(3) $$HR\left(\%\right)=\frac{\left(A_{t}-A_{nc}\right)}{\left(A_{pc}-A_{nc}\right)}\times 100\%$$

where A_t_, A_pc_, and A_nc_ are the absorbances of the test sample, positive control, and negative control, respectively. Each sample was measured in triplicate, and the average value was finally obtained, with error bars recorded.

## 3. Results

### 3.1. Synthesis and Characterization of the Cur-PU

**Figure 1:**
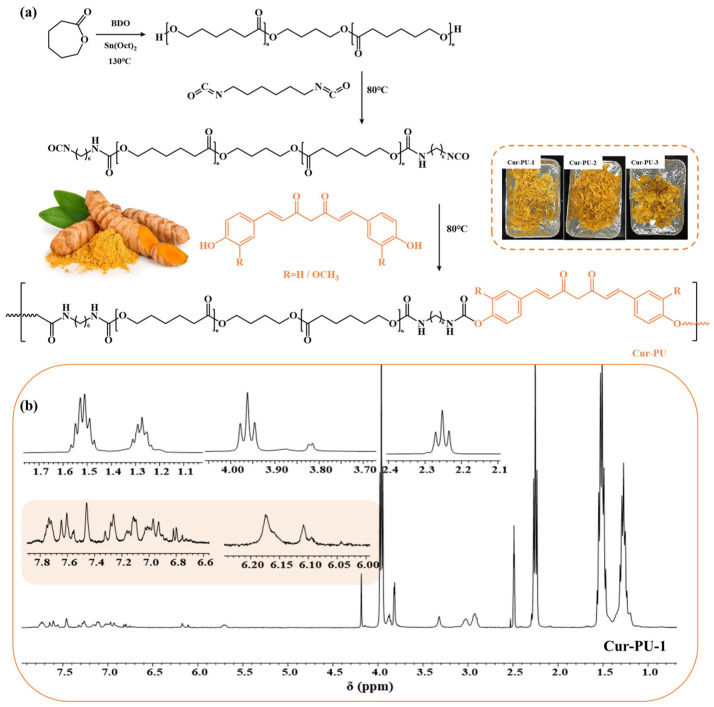
The structure and synthesis of Cur-PU (**a**), The ^1^H NMR spectra of Cur-PU-1 (**b**).

**Figure 2:**
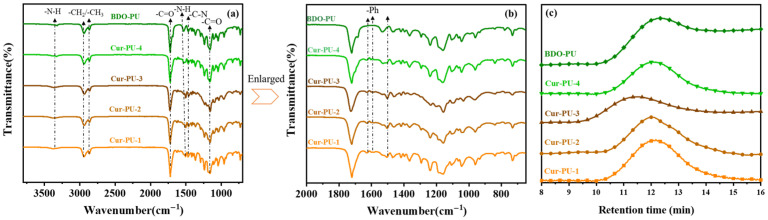
The FTIR spectra (**a**,**b**) and GPC traces (**c**) of Cur-PU and BDO-PU samples.

The synthesis pathway of the biodegradable polyurethane (Cur-PU) is depicted in Figure 1a. Initially, hydroxyl-terminated poly(ε-caprolactone) homopolymer (HO-PCL-OH) was prepared using BDO as an initiator and Sn(Oct)_2_ as the catalyst. Subsequently, Cur-PU was synthesized via polyaddition reaction between the hydroxyl groups in Cur and the isocyanate groups of the terminated prepolymer, where the prepolymer was obtained via the reaction of HO-PCL-OH with isocyanate, and the molar ratio of the reactants charged for HO-PCL-OH, isocyanate, and Cur is 1:2:1. A control sample (BDO-PU) was also prepared with 1,4-butanediol as the chain extender for comparative analysis. To investigate the substituent effects on polyurethane crystallization behavior, Cur-PU-4 was additionally synthesized with bisdemethoxycurcumin as the chain extender. Structural characterization of the as-synthesized polymer was confirmed by ^1^H NMR (Figure 1b) and FTIR spectra (Figure 2a,b). Signal assignments in the ^1^H NMR spectra were confirmed by comparison with the literature data for analogous homopolymer characterizations [35]. Specifically, the resonance signals between 6.0 and 7.8 ppm in ^1^H NMR were attributed to the aromatic protons in the curcumin moiety [36]. The absence of the characteristic –NCO stretching vibration at 2250–2270 cm^−1^ in the FTIR spectrum indicated complete consumption of isocyanate groups during polymerization, confirming the formation of carbamate linkages. In particular, the peaks between 1723 and 1731 cm^−1^ were attributed to different C=O stretching bands [37]. The intense and sharp peak at 1535 cm^−1^ corresponded to the combined C–N–H deformation vibrations of urethane groups (amide I band), while the absorption at 3360 cm^−1^ was associated with N–H stretching vibrations of urethane moieties [38]. Further analysis revealed a characteristic enol-carbonyl stretching vibration of curcumin at approximately 1630 cm^−1^. The broad absorption region between 1600 and 1400 cm^−1^ was assigned to C–H stretching vibrations of aromatic rings, with specific bands at 1601 cm^−1^ and 1508 cm^−1^ attributed to benzene-ring stretching, and C=O and C–C vibrations within the curcumin unit [30]. These results confirm the successful incorporation of curcumin into the polyurethane backbone. Gel permeation chromatography (GPC) analysis showed that the number-average molecular weight (*M*_n_) of Cur-PU ranged from 49 to 52 kDa, which is higher than that of its precursors, with relatively narrow polydispersity indexes (Ð ≤ 1.7, Figure 2c and Table S1). Collectively, the results from ^1^H NMR, FTIR, and GPC provide conclusive evidence for the successful synthesis of the target Cur-PU copolymers, demonstrating the effectiveness of the proposed synthetic route and structural design.

### 3.2. Thermal Behavioral and Crystallinity Properties of the Cur-PU

**Figure 3:**
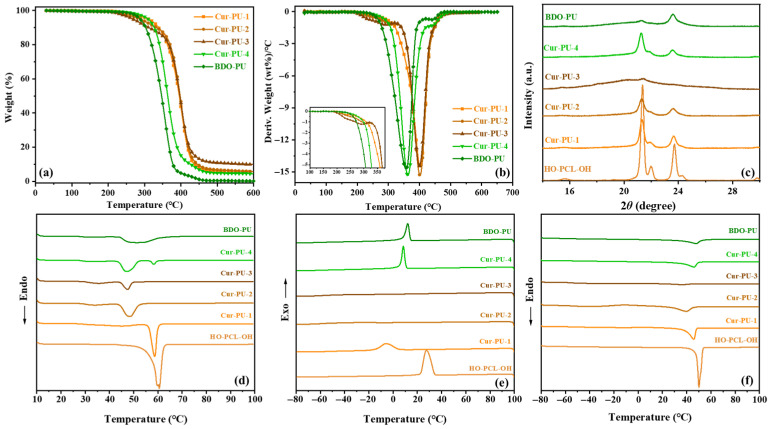
Representative TG (**a**) and DTG (**b**) curves. X-ray diffraction (XRD) spectra (**c**). DSC thermograms: the first heating scans (**d**), the first cooling scans (**e**), and the second heating scans (**f**) of Cur-PU and BDO-PU sample.

The thermal properties of Cur-PU, BDO-PU, and HO-PCL-OH prepolymer were investigated by DSC and TGA, as summarized in Figure 3 and Tables S2 and S3. The TGA and DTG curves of all samples are shown in Figure 3a,b. The results show that the initial decomposition temperature (*T*_5%_) and the maximum temperature (*T*_max_) of all polyurethanes are higher than 250 °C, and both the *T*_50%_ and *T*_max_ of Cur-PU are higher than those of BDO-PU, indicating that the incorporation of curcumin or bisdemethoxycurcumin is beneficial for enhancing the thermal stability of polyurethanes. As the curcumin content increases, the *T*_max_ only dropped slightly despite a significant decrease in the *T*_5_% of the Cur-PU material. Unlike with Cur-PU-4 and BDO-PU, the thermal degradation of the Cur-PU-1, Cur-PU-2, and Cur-PU-3 samples occurs in two stages. It is hypothesized that during the thermal degradation process, the aromatic structure of curcumin can inhibit the further cleavage of the main chain through the conjugated stabilization effect. Additionally, it may promote the formation of a carbon layer, which acts as a physical barrier, thereby exhibiting a secondary stabilizing effect.

For all samples, the first heating scans of DSC curves exhibit distinct melting peaks (Figure 3d), indicating their semicrystalline nature. The melting temperature (*T*^1^_m_) is lower than that of HO-PCL-OH (Table S2). With the increase in hard-segment content in polyurethane, the *T*^1^_m_ of Cur-PU decreases from 58.55 to 47.47 °C. This is primarily attributed to the enhanced intermolecular hydrogen bonding from increased hard-segment content, which partially restricts the movement of HO-PCL-OH chains. The highly hindered benzene ring structure in Cur disrupts the partial crystallization of polyurethane matrix, resulting in a lower melting temperature in Cur-PU-2 and Cur-PU-3 compared to BDO-PU. Based on the melting enthalpy (ΔH^1^_m_) from first heating scans, the crystallinity (χ_c_) was calculated to range from 5.33 to 32.66%, depending on the Cur content. Compared to HO-PCL-OH (56.96% of χ_c_) and BDO-PU (28.25% of χ_c_), the decrease in χ_c_ for Cur-PU is attributed to the presence of Cur units, which hinder the close and orderly packing of crystalline HO-PCL-OH chains. However, no crystalline peak was observed for Cur-PU-2 and Cur-PU-3 during the first cooling scans of DSC curves (Figure 3e), except for Cur-PU-1, further confirming the inhibited crystallization of HO-PCL-OH upon curcumin-unit incorporation. Interestingly, distinct melting peaks were observed for Cur-PU-1 and Cur-PU-2 in the second heating scans of DSC curves (Figure 3f). Both the crystallization temperature (*T*_c_) and Tm exhibited a similar decreasing trend with increasing curcumin incorporation, which is consistent with the XRD results (Figure 3c). From the XRD pattern of HO-PCL-OH, three intense diffraction peaks can be observed at Bragg angles 2θ = 21.4°, 22.1°, and 23.8°, which are attributed to the diffraction from the (110), (111), and (200) lattice planes of semi-crystalline PCL [39,40], respectively. However, these three diffraction peaks gradually disappear with the increase in Cur units, further verifying that the large steric hindrance benzene ring structure in curcumin indeed restricts the crystallization of HO-PCL-OH. Additionally, with the increase in curcumin content, the glass transition temperature (*T*_g_) of the samples gradually decreases from −54.46 °C to −56.58 °C, and this decrease may be attributed to the enhancement of chain mobility by curcumin units.

### 3.3. Mechanical Properties and Shape-Memory Behavior of the Cur-PU Films

**Figure 4:**
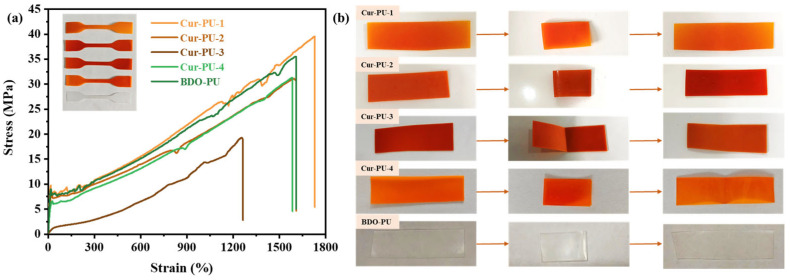
The mechanical performances (**a**) and shape memory (**b**) of Cur-PU film.

The mechanical properties of Cur-PU and BDO-PU samples were evaluated using an Instron Universal Testing machine. Samples with a thickness of approximately 0.5 mm were prepared via solution evaporates. The test results are shown in Figure 4a and summarized in Table S4.

For the Cur-PU samples, both tensile strength and hardness are lower than those of the BDO-PU samples. Although the increase in the hard-segment content of Cur-PU leads to enhanced intermolecular hydrogen bonding, which could elevate the hardness of the polyurethane material, the large steric hindrance of the benzene ring structure in curcumin severely hinders the crystallization of HO-PCL-OH. This significantly reduces both the crystallinity and crystallization rate of Cur-PU. Consequently, the strength and hardness of Cur-PU decrease with the increase in the content of Cur units. Notably, while other Cur-PU and BDO-PU samples exhibit thermoplastic characteristics, Cur-PU-3 displays elastomeric properties due to its lower crystallinity and slower crystallization kinetics. It exhibits no distinct yield point in the tensile curve and has the lowest tensile strength and hardness (19.299 MPa and 65 HA). However, both Cur-PU and BDO-PU samples exhibit excellent shape-memory functionality. As shown in Figure 4b, excluding the Cur-PU-3, both the R_f_ and R_r_ of other samples exceeded 95%. In particular, the Cur-PU-1 exhibits exceptional shape-memory functionality. In Cur-PU samples, the HO-PCL-OH soft segments endow flexibility and reversible deformation capacity, enabling the fixation and recovery of temporary shapes. The hard segments formed by the reaction of isocyanates with chain extenders act as physical crosslinking points or crystalline domains, maintaining the shape of the polyurethane. Thus, the soft (reversible phase) and hard segments (fixed phase) form a microphase-separated structure via hydrogen bonding or crystallization. Increasing the Cur units enhances the proportion of hard segments in the polyurethane, improving shape-fixing ability but reducing recovery ability in shape-memory functionality.

### 3.4. pH Responsiveness of the Cur-PU

**Figure 5:**
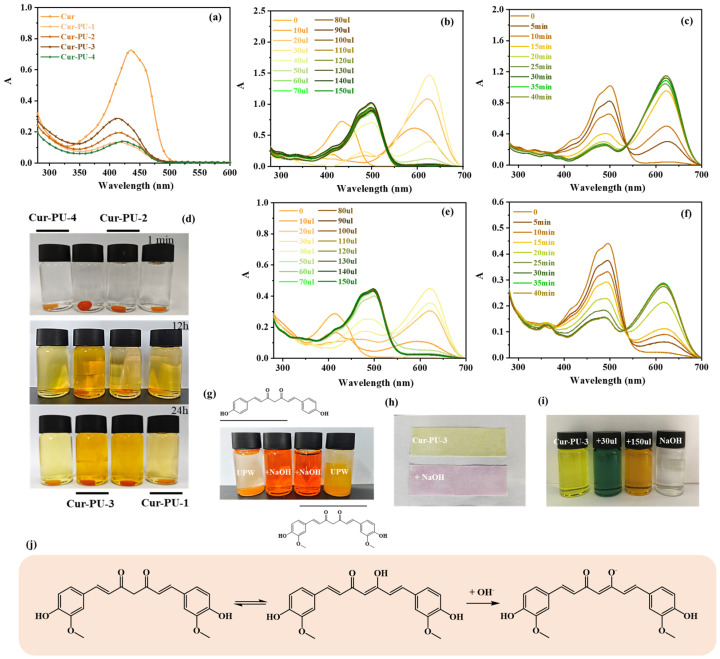
The absorption spectra of Cur-PU and Cur in DMSO (**a**). Cur solution with different volumes of NaOH solution (NaOH: 0.1 mol/L) (**b**). Cur solution with 100 μL of NaOH solution for different settling times at room temperature (NaOH: 0.1 mol/L) (**c**). Cur-PU film after soaking in NaOH solution for 0 min, 1 min, and 24 h (NaOH: 0.1 mol/L) (**d**). Cur and bisdemethoxycurcumin dissolved in NaOH solution and UPW (**g**). The absorption spectra of Cur-PU-3 solution with different volumes of NaOH solution (NaOH: 0.1 mol/L) (**e**). The absorption spectra of Cur-PU-3 solution with 150 μL of NaOH solution for different settling times at room temperature (NaOH: 0.1 mol/L) (**f**). Samples stained with Cur-PU-3 solution and samples soaked in NaOH solution again (NaOH: 0.1 mol/L) (**h**). Images captured of the Cur-PU-3 with 30 μL, 150 μL of NaOH solution (NaOH: 0.1 mol/L) (**i**). The schematic diagram of tautomeric structure (**j**).

Owing to the introduction of Cur, all solid polyurethane samples exhibited an orange-yellow hue (Figure 4a). Thus, UV-Vis absorption spectroscopy was employed to characterize the UV absorption spectra of curcumin-modified polyurethane samples, as depicted in Figure 5 and Table S5. All Cur-PU samples exhibited UV absorption peak positions similar to those of Cur, and the absorbance of their DMSO solutions was lower than that of Cur due to the limited Cur unit in the polyurethane. Conversely, with increasing Cur units in the polyurethane, the absorbance increased from 0.135 to 0.288. Cur-PU-4 also exhibited UV absorption spectra similar to those of Cur and Cur-PU, with absorbance comparable to that of Cur-PU-1 at the same concentration (A: 0.14).

It was also observed that upon the addition of different volumes of NaOH solution, the color of the Cur-PU solution shifted from orange-yellow to dark green and finally to orange (Figure 5i). For comparative analysis, the color changes in Cur and Cur-PU upon NaOH solution addition were investigated. All Cur-PU solutions showed similar color changes to those of Cur upon hydroxide-ion addition (Figure 5 and Figures S1–S3). As shown in Figure 5e, upon adding 40 μL of NaOH solution, the UV absorption of Cur-PU-3 solution showed a distinct peak at 622 nm, with an absorbance of up to 0.357, higher than the original solution. Upon the addition of 150 μL of NaOH solution, the absorption peak at 622 nm gradually diminished, and a new absorption peak emerged at 495 nm with an absorbance of 0.444. Subsequent addition of hydroxide ions induced no changes in the absorption peak position or absorbance.

However, when the Cur-PU-3 solution with 140 μL of NaOH solution added was stored at room temperature for different durations, it was observed that the solution gradually turned dark green. As shown in Figure 5f, with increasing storage time at room temperature, the absorption peak of the Cur-PU-3 solution at 495 nm gradually diminished, while the peak at 615 nm reappeared. Eventually, the solution transformed into a dark green color, and all Cur-PU samples showed this characteristic (Figures S1–S3 and Tables S8–S10). Leveraging this characteristic, a sample strip dipped in Cur-PU-3 solution was soaked in NaOH solution for 3 s and then removed. It was found that the original yellow-green sample strip turned orange-pink (Figure 5h), analogous to the aforementioned phenomenon.

Given the predominantly solid-state existence of Cur-PU materials in practical applications, the color changes in Cur-PU films upon immersion in sodium hydroxide solution for varying durations were systematically investigated (Figure 5d). As shown in Figure 5d, the as-prepared Cur-PU film exhibited an orange hue. After 1 min of immersion in sodium hydroxide solution, no discernible color transformation was observed in either the Cur-PU film or the sodium hydroxide solution. However, after 12 h of immersion, the Cur-PU film underwent slight color fading, while the sodium hydroxide solution transitioned from colorless to orange-yellow, with the Cur-PU-3 sample demonstrating the most pronounced chromatic change. Upon further immersion extended to 24 h, the intensity of the solution’s orange-yellow color deepened. Notably, a higher concentration of the sodium hydroxide solution accelerated the rate and intensified the extent of color change in the Cur-PU films during immersion. This characteristic is consistent with the intrinsic property of Cur, which is insoluble or slightly soluble in water but highly soluble in sodium hydroxide solution (Figure 5g). Therefore, the pH-responsive color changes in Cur-PU films hold promise for applications in tracking, monitoring, and intelligent response recognition systems.

According to two literature reports [41,42], Cur predominantly exists in the keto-enol form in alkaline media, with the absorption peak at approximately 420 nm originating from the π-π* transition of the keto-enol Cur. The addition of hydroxide ions reacts with the -OH groups in the keto-enol structure, gradually forming O^−^ and leading to the dynamic equilibrium illustrated in Figure 5j. Thus, the Cur-PU solution exhibits distinct color changes upon the addition of varying volumes of NaOH solution.

### 3.5. Hydrolytic Degradation of the Cur-PU Films

**Figure 6:**
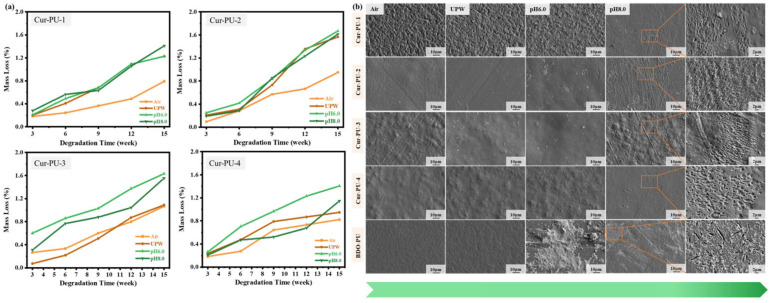
The weight loss of Cur-PU after degradation in air, UPW, and pH6.0 and pH8.0 phosphate buffer solution for 3, 6, 9, 12, and 15 weeks (**a**). The SEM photographs of Cur-PU after degradation in air, UPW, and pH6.0 and pH8.0 phosphate buffer solution for 15 weeks (**b**).

To systematically investigate the hydrolytic degradation behavior of Cur-PU, samples were prepared as 10 × 10 mm square films and subjected to a 15-week degradation experiment in four media, namely air, UPW, and pH 6.0 and pH 8.0 phosphate buffer solution, under controlled conditions of 25 °C and 60% relative humidity. During the experiment, samples were retrieved every three weeks and dried to constant weight, while SEM was employed to characterize surface morphological changes (Figure 6).

As shown in Figure 6, the mass loss of Cur-PU samples in all media remained below 2%. This can be attributed to the hydrophobic benzene ring moieties in the molecular structure of curcumin [43,44]. This structural feature increased the contact angle of Cur-PU compared to BDO-PU, endowing the material with stronger hydrophobicity (Figure S4). Consequently, the mass loss rates of Cur-PU-1 and Cur-PU-4 were lower than those of BDO-PU. However, the degradation rates of Cur-PU-2 and Cur-PU-3 are faster than those of Cur-PU-1 and Cur-PU-4. On the one hand, although the water contact angles of Cur-PU-2 and Cur-PU-3 are larger than that of BDO-PU, they exhibit lower crystallinity and slower crystallization rates (especially for the Cur-PU-3 sample). On the other hand, Cur-PU-2 and Cur-PU-3 contain more Cur units, and HO^−^ in the alkaline medium will undergo certain interactions with Cur in the molecular chains (as Figure 5j), thereby accelerating the degradation process. In weakly acidic or alkaline media, the degradation rate of polyurethane samples was slightly higher than in UPW and air environments, a trend further confirmed by surface morphology analysis. As shown in Figure 6b, after 15 weeks of degradation in pH 8.0 phosphate buffer, apparent etched pores appeared on the surfaces of Cur-PU samples, with Cur-PU-2 and Cur-PU-3 exhibiting more densely distributed pores. Such structural characteristics facilitated the penetration of water molecules into the interior of the material, thereby accelerating the degradation process. In conclusion, the hydrolytic degradation of Cur-PU materials exhibits a degradation pattern similar to that of pure PCL, specifically a surface erosion-dominated degradation mode, with a slow degradation rate.

### 3.6. In Vitro and in Vivo Biocompatibility of the Cur-PU Films

**Figure 7:**
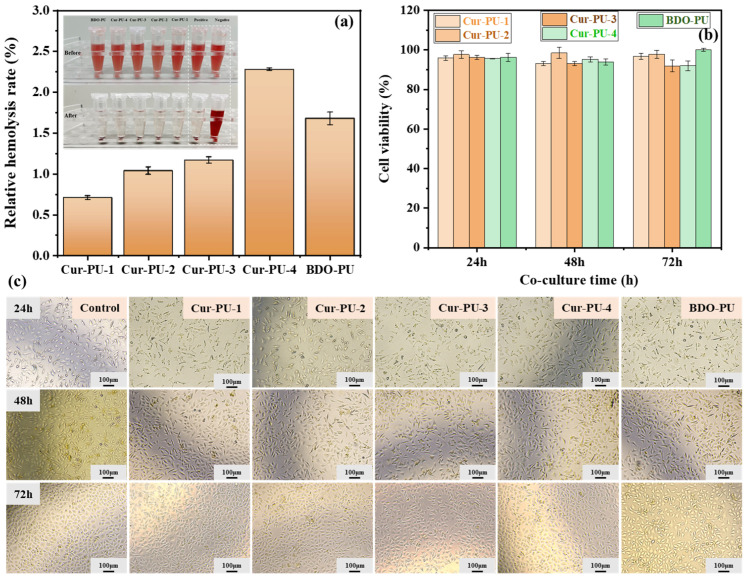
The photo of hemolysis test (**a**). Cell viability of L929 cells cultured for 24 h, 48 h, and 72 h in extracts of the of Cur-PU and BDO-PU films (**b**). Microscopic images of L929 cells cultured for 24 h, 48 h, and 72 h in extracts of the control group and Cur-PU (**c**).

Mouse fibroblasts (L929 cells), a key cell type in wound repair, were used to evaluate the in vitro biocompatibility of Cur-PU and BDO-PU. CCK-8 assay results revealed that the cell viability of 50% (*v*/*v*) polyurethane extract exceeded 90% (Figure 7b and Table S11). With prolonged co-culture time, cell proliferation rates increased, exhibiting a growth pattern comparable to that of the control group (Figure 7c), indicating the good biocompatibility of Cur-PU materials.

To further assess the histocompatibility of the polyurethanes, the hemolysis ratio of red blood cells (RBCs) was evaluated using rabbit blood. All polyurethanes exhibited hemolysis ratios ranging from 0.71 to 2.28%, lower than those of the deionized-water group (100%). Figure 7a shows that the supernatants for all the polyurethane groups were nearly colorless and transparent, similar to those of the PBS group with a hemolysis ratio of 0, confirming that most red blood cells remained intact in the presence of the polyurethanes. In sharp contrast, the supernatant of the deionized-water group was deeply red, indicating a high hemolysis ratio. In summary, these results confirm the good hemocompatibility of the Cur-PU and BDO-PU materials.

## 4. Conclusions

Using HO-PCL-OH as the soft segment and curcumin as the chain extender, a polyurethane material (Cur-PU) with good biocompatibility was synthesized through a one-pot, two-step synthesis approach. The effects of different amounts of Cur content on the thermal stability, crystalline properties, mechanical behaviors, hydrolytic degradability, and biocompatibility of the polyurethane were systematically investigated. Experimental results showed that as the curcumin content increased, the *T*_m_ and ∆H_m_ dropped from 58.55 °C and 40.818 J/g to 47.47 °C and 11.407 J/g, respectively. The χ_c_ decreased from 32.66% to 5.33%. Notably, the tensile strength and hardness decreased from 35.5 MPa and 88 HA to 19.3 MPa and 65 HA. These observations indicate that the sterically hindered benzene ring structure of curcumin partially hinders the crystallization of HO-PCL-OH, leading to lower crystallinity and slower crystallization kinetics in Cur-PU, which in turn affect the material’s strength and hardness. Therefore, precise tuning of the mechanical properties of polyurethane can be achieved by adjusting the soft–hard segment ratio. Additionally, Upon the addition of varying volumes of NaOH solution to all Cur-PU solutions, the solution color transitioned from initial yellow-green to dark green and finally to orange-yellow, enabling sensitive pH-responsive. The mass loss of Cur-PU films remained below 2% after hydrolytic degradation, exhibiting a surface-erosion degradation pattern comparable to that of BDO-PU. Results from CCK-8 cytotoxicity assays and relative red blood cell hemolysis tests confirmed that all Cur-PU and BDO-PU samples have good biocompatibility. Given the limitations of the current experiment, in future work, we will simulate the in vivo degradation environment to conduct a study on the degradation process of Cur-PU materials in organisms and the toxicity of their degradation products, so as to explore the potential of Cur-PU materials for biomedical applications such as drug delivery, wound dressings, and long-term implanted medical devices.

## Data Availability

The original contributions presented in this study are included in the article. Further inquiries can be directed toward the corresponding author.

## Supplementary Materials

The following supporting information can be downloaded at https://www.mdpi.com/article/10.3390/biom15081070/s1, Figure S1: Cur-PU-1 solution with different volumes of NaOH solution (a). Cur-PU-1 solution with different volumes of NaOH solution for different settling times at room temperature (b). (Cur-PU-1: 0.055 mg/mL; solvent: DMSO; NaOH solution: 0.1 mol/L); Figure S2: Cur-PU-2 solution with different volumes of NaOH solution (a). Cur-PU-2 solution with different volumes of NaOH solution for different settling times at room temperature (b). (Cur-PU-2: 0.055 mg/mL; solvent: DMSO; NaOH solution: 0.1 mol/L); Figure S3: Cur-PU-4 solution with different volumes of NaOH solution (a). Cur-PU-4 solution with different volumes of NaOH solution for different settling times at room temperature (b). (Cur-PU-4: 0.055 mg/mL; solvent: DMSO; NaOH solution: 0.1 mol/L); Figure S4: The water contact angle of Cur-PU film. Figure S5: The weight loss of BDO-PU; Table S1: The chemical composition and molecular characteristics of Cur-PU and BDO-PU sample; Table S2: The TG results of Cur-PU and BDO-PU; Table S3: The DSC results of Cur-PU, BDO-PU, and HO-PCL-OH prepolymer; Table S4: Mechanical performances and water contact angle of Cur-PU samples; Table S5: Detailed data of the ratio (R_f_) and recovery ratio (R_r_) of Cur-PU samples; Table S6: The UV-Vis absorption data of all Cur-PU samples and the Cur sample at room temperature; Table S7: The UV absorption data of Cur solution with 100 μL of NaOH solution for different settling times at room temperature (Cur: 0.0055 mg/mL; solvent: DMSO; NaOH solution: 0.1 mol/L); Table S8: The UV absorption data of Cur-PU-3 solution with 150 μL of NaOH solution for different settling times at room temperature (Cur-PU-3: 0.055 mg/mL; solvent: DMSO; NaOH solution: 0.1 mol/L); Table S9: The UV absorption data of Cur-PU-1 solution with 150 μL of NaOH solution for different settling times at room temperature (Cur-PU-1: 0.055 mg/mL; solvent: DMSO; NaOH solution: 0.1 mol/L); Table S10: The UV absorption data of Cur-PU-2 solution with 150 μL of NaOH solution for different settling times at room temperature (Cur-PU-2: 0.055 mg/mL; solvent: DMSO; NaOH solution: 0.1 mol/L); Table S11: The UV absorption data of Cur-PU-4 solution with 150 μL of NaOH solution for different settling times at room temperature (Cur-PU-4: 0.055 mg/mL; solvent: DMSO; NaOH solution: 0.1 mol/L); Table S12: The results of Cell viability of L929 cells cultured for 24 h, 48 h, and 72 h in extracts of the Cur-PU and BDO-PU film and the hemolysis test of Cur-PU and BDO-PU.
