# Supplementary material for: Design and Characterization of Curcumin-Modified Polyurethane Material with Good Mechanical, Shape-Memory, pH-Responsive, and Biocompatible Properties
Source: Biomolecules. 2025 Jul 24;15(8):1070. doi: 10.3390/biom15081070 (PMC12383527; doi:10.3390/biom15081070)
Supplement: Supplementary file 1 [file biomolecules-15-01070-s001.zip › biomolecules-3745805-supplementary.pdf]

Supporting Information

Design and Characterization of Curcumin-Modified Polyurethane Material with good Mechanical, Shape-Memory, pH-Responsive, and Biocompatible Properties

Man Wang†[1*], Hongying Liu†[4], Wei Zhao[1], Huafen Wang[1], Yuwei Zhuang[1], Jie Yang[1], Zhaohui Liu[1], Jing Zhu[1], Sichong Chen[2], Jinghui Cheng[3,*]

[1] High & New Technology Research Center of Henan Academy of Sciences, No. 56 Hongzhuan Road, Zhengzhou 450002, P.R. China; manmanwang202309@hnas.ac.cn (M.W.); ZhaoWei@hnas.ac.cn; (W.Z.); wanghuafen_2025@hnas.ac.cn (H.-F.W.); yuweizhuang0218@163.com (Y.-W.Z); YangJie@hnas.ac.cn; (J.Y.); zjlsl7443@hnas.ac.cn; (J.Z.); LiuZhaohui@hnas.ac.cn; (Z.-H.L.);

[2] The Collaborative Innovation Center for Eco-Friendly and Fire-Safety Polymeric Materials (MoE), National Engineering Laboratory of Eco-Friendly Polymeric Materials (Sichuan), College of Chemistry, State Key Laboratory of Polymer Materials Engineering, Sichuan University, Chengdu 610064, China; chensichong@scu.edu.cn (S.-C.C);

[3] Key Laboratory of Chemo/Biosensing and Detection of Xuchang, Key Laboratory of Micro-Nano Materials for Energy Storage and Conversion of Henan Province, Henan Joint International Research Laboratory of Nanomaterials for Energy and Catalysis, College of Chemical and Materials Engineering, Xuchang University, 461000, P.R. China. 12018003@xcu.edu.cn (J.-H.C.);

[4] West China School of Nursing, Sichuan University, No.37, Guoxue Alley, Chengdu, 610041, Sichuan Province, China. liuhongying@scu.edu.cn (H.-Y.L.);

* Correspondence: manmanwang202309@hnas.ac.cn (M.W.); 12018003@xcu.edu.cn (J.-H.C.);

Figure S1: Cur-PU-1 solution with different volumes of NaOH solution (a). Cur-PU-1 solution with different volumes of NaOH solution for different settling times at room temperature (b). (Cur-PU-1: 0.055 mg/mL; solvent: DMSO; NaOH solution: 0.1 mol/L.)

Figure S2: Cur-PU-2 solution with different volumes of NaOH solution (a). Cur-PU-2 solution with different volumes of NaOH solution for different settling times at room temperature (b). (Cur-PU-2: 0.055 mg/mL; solvent: DMSO; NaOH solution: 0.1 mol/L.)

Figure S3: Cur-PU-4 solution with different volumes of NaOH solution (a). Cur-PU-4 solution with different volumes of NaOH solution for different settling times at room temperature (b). (Cur-PU-4: 0.055 mg/mL; solvent: DMSO; NaOH solution: 0.1 mol/L.)

Figure S4: The water contact angle of Cur-PU film.

Figure S5: The weight loss of BDO-PU.

Table S1: The chemical composition and molecular characteristics of Cur-PU and BDO-PU.

Table S2: The TG results of Cur-PU and BDO-PU.

Table S3: The DSC results of Cur-PU, BDO-PU, and HO-PCL-OH prepolymer.

Table S4: Mechanical performances and water contact angles of Cur-PU samples.

Table S5: Detailed data of the ratio (R_f) and recovery ratio (R_r) of Cur-PU samples.

Table S6: The UV-Vis absorption data of all Cur-PU samples and the Cur sample at room temperature.

Table S7: The UV absorption data of Cur solution with 100 μL of NaOH solution for different settling times at room temperature (Cur: 0.0055 mg/mL; solvent: DMSO; NaOH solution: 0.1 mol/L).

Table S8: The UV absorption data of Cur-PU-3 solution with 150 μL of NaOH solution for different settling times at room temperature (Cur-PU-3: 0.055 mg/mL; solvent: DMSO; NaOH solution: 0.1 mol/L).

Table S9: The UV absorption data of Cur-PU-1 solution with 150 μL of NaOH solution for different settling times at room temperature (Cur-PU-1: 0.055 mg/mL; solvent: DMSO; NaOH solution: 0.1 mol/L).

Table S10: The UV absorption data of Cur-PU-2 solution with 150 μL of NaOH solution for different settling times at room temperature (Cur-PU-2: 0.055 mg/mL; solvent: DMSO; NaOH solution: 0.1 mol/L).

Table S11: The UV absorption data of Cur-PU-4 solution with 150 μL of NaOH solution for different settling times at room temperature (Cur-PU-4: 0.055 mg/mL; solvent: DMSO; NaOH solution: 0.1 mol/L).

Table S12: The results of cell viability of L929 cells cultured for 24 h, 48 h, and 72 h in extracts of the Cur-PU and BDO-PU films and the hemolysis test of Cur-PU and BDO-PU.

Table S1. The chemical composition and molecular characteristics of Cur-PU and BDO-PU samples.

Sample	n^a_{Cur}(%)	φ^b_{Cur}(%)	φ^b_{PCL}(%)	M^c_n(kDa)	M^c_w(kDa)	Đ
HO-PCL-OH	0	0	100	2.427	3.246	1.337
Cur-PU-1	1.01	3.157	96.84	50.513	75.079	1.486
Cur-PU-2	2.01	6.180	93.82	49.794	78.070	1.568
Cur-PU-3	3.02	9.076	90.12	51.109	89.738	1.756
Cur-PU-4	1.00	2.66	97.34	53.590	81.384	1.519
BDO-PU	0	0	100	51.713	75.332	1.457

Prepolymer: HO-PCL-OH: M_w ~ 3000g/mol;

[a] n_{Cur}(%) measured by ^{1}H NMR of the quenched solution; [b] φ_{Cur}(%) calculate by (1).

$\varphi_{Cur}(\%) = (n_{Cur}*M_{Cur})/(n_{Cur}*M_{Cur}+n_{CL}*M_{CL})$ (1).

[C] Number-average molecular weight (M_n) and dispersity index (Đ=M_w/M_n), determined by gel permeation chromatography (GPC) at 30 °C in DMF.

Table S2. The TG results of Cur-PU and BDO-PU.

Sample	$T_{5\%}$ (°C)	$T_{50\%}$ (°C)	T_{max}(°C)
BDO-PU	288.1	346.83	356.51
Cur-PU-1	309.9	395.76	402.44
Cur-PU-2	287.9	397.64	401.45
Cur-PU-3	267.9	397.32	399.95
Cur-PU-4	309.9	363.58	362.44

Table S3. The DSC results of Cur-PU, BDO-PU, and HO-PCL-OH prepolymers.

Sample	T_g (°C)	T^1_m (°C)	H^1_m (J/g)	T^2_m (°C)
HO-PCL-OH	-60.58	60.57	96.947	50.14
BDO-PU	-57.01	51.37	38.905	48.10
Cur-PU-1	-54.46	58.55	40.818	45.71
Cur-PU-2	-56.08	48.50	33.179	39.79

Cur-PU-3	-56.58	47.47	11.407	36.32
Cur-PU-4	-57.78	47.28	28.689	46.22

Sample	H^2_m (J/g)	T^1_c (℃)	H^1_c(J/g)	$\chi_{C,PCL}$(%)
HO-PCL-OH	79.169	27.42	75.829	56.96
BDO-PU	29.542	12.22	34.329	28.25
Cur-PU-1	43.964	-6.00	34.794	32.66
Cur-PU-2	29.522	/	/	22.64
Cur-PU-3	6.7388	/	/	5.33
Cur-PU-4	30.624	8.66	33.847	22.63

T^1_m determined from the first heating scan.

T^1_c determined from the first cooling scan.

T^2_m and T^2_c of the samples determined from the second heating scan.

The degree of crystallinity (χ_c) calculated from the second heating scan.

Table S4. Mechanical performances and water contact angles of Cur-PU and BDO-PU samples.

Sample	Tensile Strength (MPa)	Elongation at Break (%)	Hardness (HA)	WCA (°)
Cur-PU-1	39.581±0.9	1729.067±17.3	88±3	91.5±0.1
Cur-PU-2	31.119±3.9	1609.764±12.34	82±2	89.0±0.1
Cur-PU-3	19.299±1.6	1262.742±9.01	65±1	100.4±0.1
Cur-PU-4	31.321±3.1	1584.424±11.21	84±2	98.8±0.4
BDO-PU	35.547±2.3	1608.516±21.11	89±3	87.0±0.1

Table S5. Detailed data of the ratio (R_f) and recovery ratio (R_r) of Cur-PU and BDO-PU samples.

Sample	R_f (%)	R_r (%)
Cur-PU-1	99.9±0.01	99.9±0.01
Cur-PU-2	98.89±0.01	97.78±0.02
Cur-PU-3	80.56±0.01	77.78±0.01
Cur-PU-4	98.33±0.01	97.22±0.02
BDO-PU	99.9±0.01	99.9±0.01

Table S6. The UV-Vis absorption data of all Cur-PU samples and the Cur sample at room temperature.

Sample	Concentration (mg/mL)	λ_{abs} (nm)	A
Cur	0.0055	436	0.726
Cur-PU-1	0.055	414	0.135
Cur-PU-2	0.055	418	0.196
Cur-PU-3	0.055	413	0.288
Cur-PU-4	0.055	421	0.14

Table S7. The UV absorption data of Cur solution with 100 μL of NaOH solution for different settling times at room temperature (Cur: 0.0055 mg/mL; solvent: DMSO; NaOH: 0.1 mol/L).

Time（min）	λ^1_{abs} (nm)	A^1	λ^2_{abs} (nm)	A^2
0	499.5	1.019	623.0	0.040
5	498.0	0.823	623.0	0.304
10	497.0	0.658	623.5	0.502
15	492.0	0.406	623.0	0.955
20	486.0	0.301	621.5	1.141
25	486.0	0.274	623.0	1.150
30	285.5	0.253	621.5	1.119
35	485.0	0.259	621.5	1.087
40	488.5	0.250	621.5	1.046

Table S8. The UV absorption data of Cur-PU-3 solution with 150 μL of NaOH solution for different settling times at room temperature (Cur-PU-3: 0.055 mg/mL; solvent: DMSO; NaOH: 0.1 mol/L).

Time（min）	λ^1_{abs} (nm)	A^1	λ^2_{abs} (nm)	A^2
0	495.0	0.444	616.0	0.020
5	493.5	0.375	616.0	0.062
10	492.5	0.332	615.5	0.090
15	492.5	0.292	615.5	0.112
20	487.5	0.229	616.0	0.214

25	484.5	0.184	615.5	0.275
30	486.5	0.157	615.5	0.287
35	485.5	0.155	615.5	0.288
40	484.5	0.155	615.5	0.282

Figure S1. Cur-PU-1 solution with different volumes of NaOH solution (a). Cur-PU-1 solution with different volumes of NaOH solution for different settling times at room temperature (b). (Cur-PU-1: 0.055 mg/mL; solvent: DMSO; NaOH: 0.1 mol/L.)

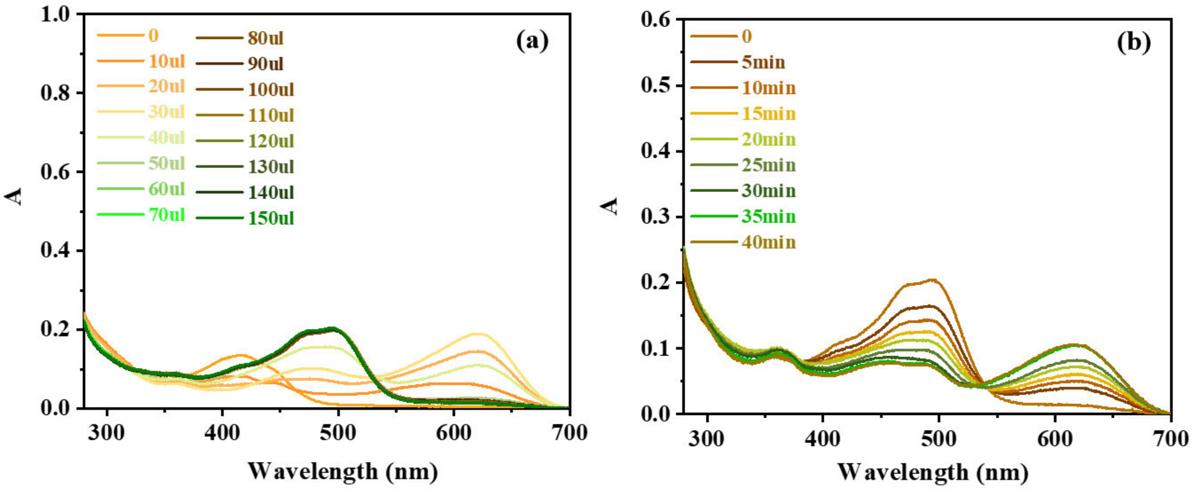

Table S9. The UV absorption data of Cur-PU-1 solution with 150 μL of NaOH solution for different settling times at room temperature (Cur-PU-1: 0.055 mg/mL; solvent: DMSO; NaOH: 0.1 mol/L).

Time（min）	λ^1_{abs} (nm)	A^1	λ^2_{abs} (nm)	A^2
0	491.0	0.204	612.0	0.014
5	491.0	0.165	612.0	0.040
10	486.5	0.143	611.5	0.049
15	486.5	0.126	612.0	0.059
20	478.0	0.113	612.5	0.072
25	482.5	0.098	619.0	0.083
30	452.5	0.086	614.5	0.105
35	456.0	0.081	619.0	0.105
40	453.5	0.078	612.0	0.106

Figure S2. Cur-PU-2 solution with different volumes of NaOH solution (a). Cur-PU-2 solution with different volumes of NaOH solution for different settling times at room temperature (b). (Cur-PU-2: 0.055 mg/mL; solvent: DMSO; NaOH: 0.1 mol/L.)

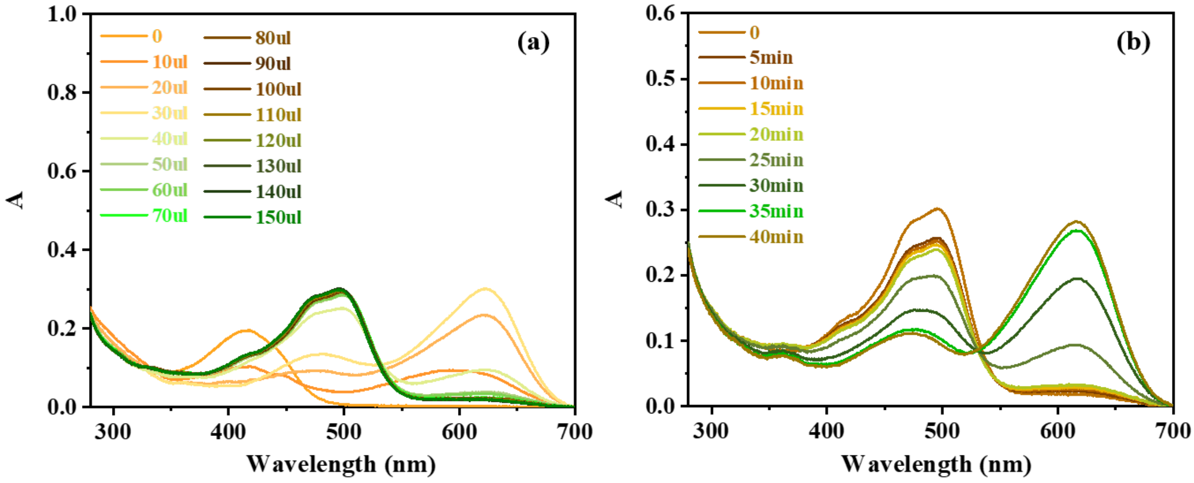

Table S10. The UV absorption data of Cur-PU-2 solution with 150 μL of NaOH solution for different settling times at room temperature (Cur-PU-2: 0.055 mg/mL; solvent: DMSO; NaOH: 0.1 mol/L).

Time（min）	λ^1_{abs} (nm)	A^1	λ^2_{abs} (nm)	A^2
0	495.5	0.302	614.5	0.018
5	492.5	0.257	614.5	0.023
10	493.0	0.253	614.5	0.026
15	493.0	0.247	614.5	0.029
20	492.5	0.239	609.5	0.034
25	492.5	0.200	614.5	0.094
30	475.0	0.147	616.0	0.195
35	477.0	0.118	615.5	0.269
40	474.5	0.112	614.5	0.281

Figure S3. Cur-PU-4 solution with different volumes of NaOH solution (a). Cur-PU-4 solution with different volumes of NaOH solution for different settling times at room temperature (b). (Cur-PU-4: 0.055 mg/mL; solvent: DMSO; NaOH: 0.1 mol/L.)

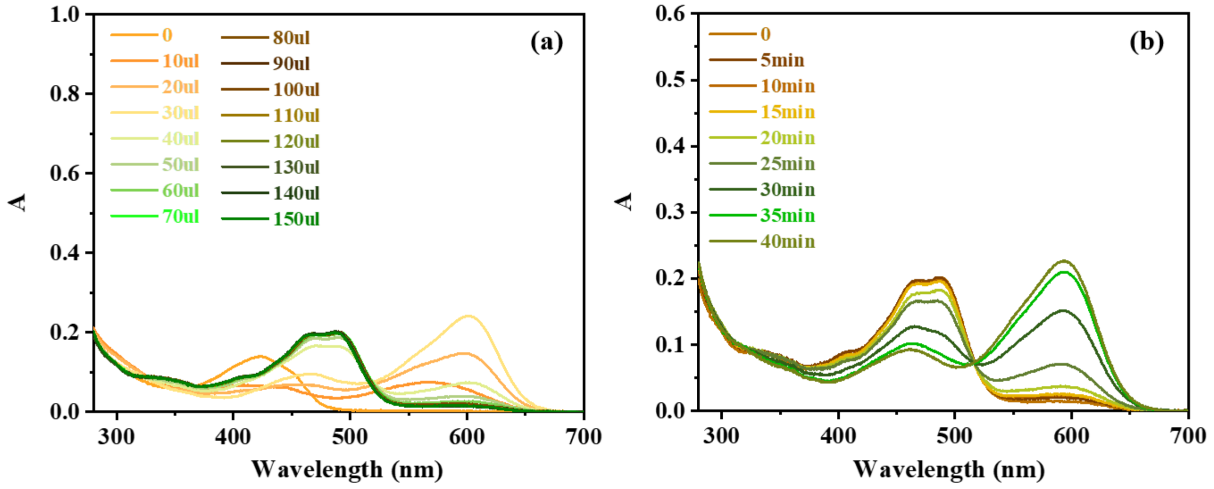

Table S11. The UV absorption data of Cur-PU-4 solution with 150 μL of NaOH solution for different settling times at room temperature (Cur-PU-4: 0.055 mg/mL; solvent: DMSO; NaOH: 0.1 mol/L).

Time（min）	λ^1_{abs} (nm)	A^1	λ^2_{abs} (nm)	A^2
0	486.0	0.202	592.5	0.015
5	485.0	0.201	593.5	0.021
10	485.0	0.198	592.5	0.025
15	485.0	0.196	592.0	0.026
20	484.5	0.183	592.5	0.037
25	485.0	0.165	592.5	0.071
30	484.0	0.119	594.5	0.152
35	483.0	0.090	592.5	0.210
40	483.0	0.079	592.5	0.227

Figure S4. The water contact angles of Cur-PU films.

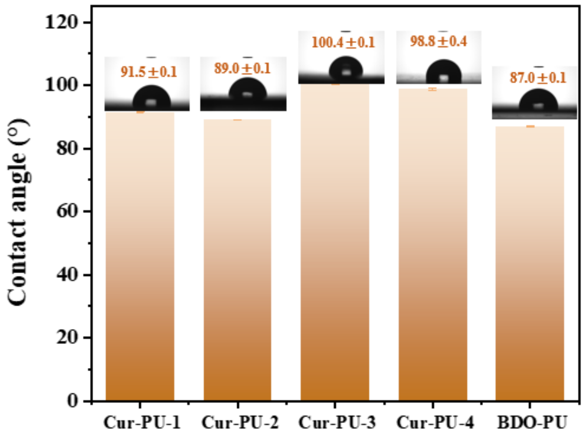

Figure S5. The weight loss of BDO-PU.

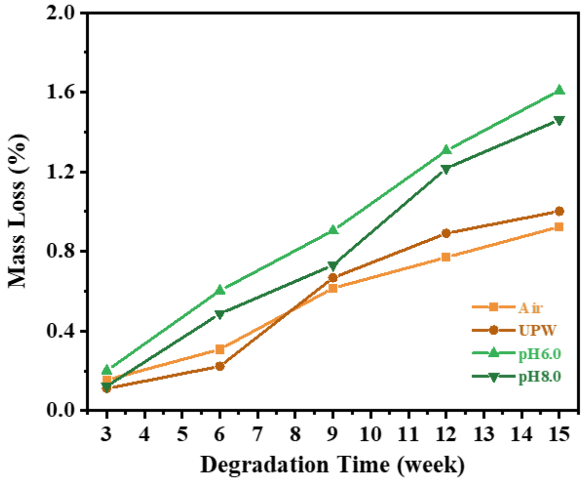

Table S12. The results of cell viability of L929 cells cultured for 24 h, 48 h, and 72 h in extracts of the of Cur-PU and BDO-PU films and the hemolysis test of Cur-PU and BDO-PU.

Sample	Cell Culture Time (h)			Relative Hemolysis Rate (%)
	24 h Cell Viability (%)	48 h Cell Viability (%)	72 h Cell Viability (%)	
Cur-PU-1	95.82%±1.73	93.05%±1.09	96.69%±1.25	0.71±0.024
Cur-PU-2	97.62%±2.03	98.45%±2.39	97.73%±2.01	1.04±0.044
Cur-PU-3	96.18%±0.36	93.08%±4.01	91.85%±0.96	1.17±0.039
Cur-PU-4	95.55%±1.87	95.13%±2.21	91.94%±0.12	2.28±0.016
BDO-PU	96.27%±1.02	93.75%±0.98	100.84%±2.09	1.68±0.077
